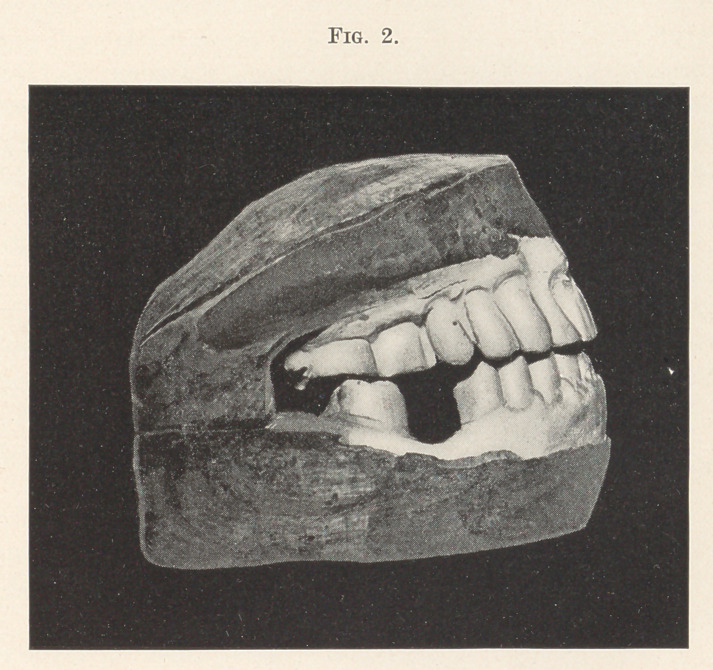# The New York Institute of Stomatology

**Published:** 1903-11

**Authors:** 

**Affiliations:** The New York Institute of Stomatology


### Reports of Society Meetings.

### THE NEW YORK INSTITUTE OF STOMATOLOGY.

A MEETING of the Institute was held at the Chelsea, No. 222 West Twenty-third Street, New York, on Tuesday evening, May 5, 1903, the president, Dr. J. Morgan Howe in the chair.

The minutes of the last meeting were read and approved.

Dr. E. A. Bogue exhibited three specimens of color—first, as it presented; second, after bleaching; and the third, seven years later—from a case in which he had treated two central incisors in 1896, and they had retained their color remarkably well.

The following method was used. The pulp-chambers and canals being unfilled, the teeth were first treated with carbolic acid, and as soon as possible the apices of the roots were filled with oxychloride of zinc, carried there on a broach wrapped with cotton. After the cement had set, the roots were cleared and considerably enlarged. The teeth were thoroughly dried with hot air, and oxalic acid full strength was put in and allowed to remain four or five minutes. The teeth were wiped out and dry chalk put in and left overnight. The next day oxalic acid was again applied, and when that had accomplished its work a twenty-five per cent. solution of pyrozone was used. The root-canals were then dried and varnished with copal dissolved in ether and filled with oxyphosphate of zinc.

Dr. J. Morgan Howe presented some models showing the restoration of teeth that had been badly worn by the stress of mastication. Two of the models are illustrated, one representing the case as it presented, and the other showing the condition after the restoration. The case was seen in 1898, when the patient complained that he was not able to eat well. The first model illustrates the condition which then obtained. (Fig. 1.) The wearing down had been very great. The upper incisors were impinging on the gum, inside of the lower incisors, and a good deal of friction on the gums resulted from mastication. It was noticeable that the teeth were entirely free from tartar before restoration, but it began

FIG. 1.

FIG. 2.

to accumulate afterwards. There was very little decay. It was a pure case of abrasion unaffected by erosion. The restoration was effected without devitalization of any pulps, and in the case of the incisors and canines consisted of a base with three or four pins entering drill-holes in the dentine, and to the periphery of this base a ferrule was soldered. Then a porcelain facing was attached, backed and built up behind with clasp gold. The molars and bicuspids were built up with solid gold cusps made of clasp-gold and solder, attached to the teeth by means of pins entering the dentine and ferrules. Phosphate cement was used for attachment. One or two of the molars were restored with a hard amalgam containing a great deal of silver. This was built around pins set with cement in drill holes in the teeth. These fillings were packed and contoured by means of temporary ferrules or ring matrices around the teeth. (Fig. 2.) Within four years the patient had ground down all these hard gold tips to such an extent that some of the facings were broken off, so that repair seemed to be demanded, and yet the abrasive force was so great that there was little prospect of permanency. In no case had any of the tips loosened from their attachments to the teeth, but the metal was worn away. This case represents the maximum of abrasive force exerted in any case Dr. Howe has seen. At the other extreme the minimum of abrasion may be represented by the case of a patient for whom he had filled a large cavity in the morsal surface of one of his lower molars with tin-foil more than twenty-five years ago. This filling, although somewhat worn, is still in good condition. Dr. Black found that the force of occlusion varied in different patients from sixty to two hundred and seventy pounds on the molar teeth, according to a report of experiments in 1895. Dr. Black stated that the condition of the peridental membrane had more to do with the exertion of this force than the muscles of mastication themselves.

Dr. Howe expressed the conviction that, notwithstanding the unfortunate failure of the operation first described, similar restorations in other mouths had been so successful that it is undoubtedly a desirable procedure.

In most mouths the restoration described would have endured all stress for many years.

Many patients exert so little abrasive force in the process of mastication that the value of tin and gutta-percha as filling-materials should by no means be overlooked, even in situations where exposed to some abrasion.

Dr. C. O. Kimball, in connection with this case, presented models of a similar case that he had treated several years ago. He had been handicapped in this case, as the patient insisted that no gold show, and objected to any artificial appliance. It was interesting in that two or three different plans were tried with different teeth. Dr. Kimball presented one model of the case after three years' wear. After two years' wear the cusps had broken away from two of the teeth and had been replaced. Of the canines, some of them were built up with gold and some with soldered gold tips as described. Around one of the molars a platinum-lined gold band had been placed, larger than the tooth at the grinding surface, to give a greater articulation, and the whole filled with amalgam. One bicuspid had been restored by means of a gold crown, carrying a shoulder that pressed against the adjoining tooth, thus bringing these two teeth together.

The secretary read a paper by Dr. L. C. Bryan, of Basel, Switzerland, entitled "Prophylaxis: Extension *for* Prevention, or Extension *of* Prevention."

(For Dr. Bryan's paper, see page 831.)

Dr. E. A. Bogue was a radical in his views, as many of the brethren knew. He strongly believed that when natural conditions were interfered with, then was the beginning of suffering; when the arrangement of the teeth was changed by extraction or by filing, so that the tuberosities of the teeth failed to come into proper contact, the deposit of tartar began. Of course the deposits did begin from other causes, but not very much, as a rule, when the mouth was healthy. This was a body of gentlemen who seldom if ever saw a healthy mouth. The mouths they came in contact with were "sick." He had in mind a gentleman, fifty-three years of age, who had never in his life used a tooth-brush, and yet whose teeth were clean and white and whose gums were in a perfectly healthy condition. It was explained by the fact that the teeth were well formed and rounded; the position of the teeth in the two arches was almost what it ought to be and the man's life had been an out-of-door life. His food had been plain and simple. He always used from one to three glasses of water to rinse his teeth. The result was that his teeth were practically self-cleansing.

The leaning forward of the posterior teeth, caused by extraction, leaves a beautiful ledge for the collection of tartar. Dr. Bogue was pleased to see that Dr. Bryan had taken up the cudgels in this direction and wished we all might.

Dr. S. E. Davenport thought the paper just read had taught Institute members that Dr. Bryan was a progressive man and one who possessed finger ability of a high order. Wielding a facile pen, he had taken to thinking very deeply upon professional subjects. Dr. Bryan was so anxious to do the best for his patients that he had for some time followed the plan of taking casts of practically every one whom he was called upon to serve to any considerable extent, the consequence being that when operating Dr. Bryan had before him not only the chart, but also the cast. The advantage of the cast over the mouth itself in studying the relation of the teeth is well understood, the cast being arranged so that the occlusion of the teeth can be observed from behind. It might seem a method entailing a great deal of trouble, but for one desiring to do the very best for his patients it had everything in its favor. There were many ways in which the plan saved a great deal of time, and made better service possible.

There was one point Dr. Davenport would like to emphasize and commend,—the use of iodine upon and between the teeth, and not merely as an adjunct to take away the green stain upon children's teeth. It would be noticed in using it that where there was any deposit whatever the iodine stained it, thus pointing out the dentist's duty and aiding him chemically in the performance of it.

Dr. H. W. Gillett was not able to get away from Dr. Bryan's first paragraph. It seemed to him a wrong assumption. Dr. Gillett did not think it would be universally admitted that there was an increase of decay of the teeth from generation to generation. It seemed to him that such a statement was not a good foundation for a paper, and he would not concede that it was the usual thing for patients coming for annual or semiannual visits to have, "for a mathematical certainty," a large number of cavities to be filled. If he found such a condition in the mouths of patients who only came *once* a year he certainly would not have a clear conscience. Either he would not be doing his whole duty or he would lack sufficient force to make his patients do theirs. Dr. Gillett felt that pyorrhœa developing in young patients constantly under his care was largely his fault. One of the points that surprised him most in Dr. Bryan's paper was the apparent difficulty made over doing the necessary *little* things about the mouth. It is just as essential that these little things be placed upon the examination chart as that actual cavities be put down. They were just as much a part of our work. He tells his patients that cleansing their teeth is the most important operation he does for them, and feels that the day is coming when the extension for prevention will be much less necessary. Within the next decade there was going to be much more preventive work done along lines already pointed out. Extension for prevention would still be necessary, however, in many cases. We will always have with us the patient who goes to the dentist once in five, seven, or eight years, and after being put in order does not expect to come for another ten years, and hopes never to come again.

Dr. Gillett was heartily in sympathy with the work Dr. Bryan had outlined. Anything we can do to prevent decay, we should do. Thoroughness in all kinds of work would help us in this direction.

Dr. Kimball's eye, too, was caught by Dr. Bryan's first phrase. The result of his observation differed from that of Dr. Bryan's. The teeth, as he saw them, were improving rather than deteriorating. He would feel rather sorry for the profession if this were not so. At the same time there was no question that we did not do as much of this preventive treatment as we should. Dr. Kimball was inclined to think, with Dr. Gillett, that we did not expect to find, in the mouths of our regular patients, a large amount of decay from year to year. He called to mind an article by Dr. Jack in which he stated that in his regular work, by his system of sending for patients whenever he chooses, he allowed in the average mouth three appointments for work each year, besides cleansing. Dr. Kimball found that this same rule applied in his practice.

He wanted to say a little about Dr. Smith's position and work. Dr. Bryan alluded to it. It was worth while to say now, for our elucidation, that while Dr. Smith makes the claim that this process of cleansing teeth with sticks and pumice, and that he is the author of this method of prophylaxis, in reality the oldest of us were children in the profession when this was taught us and taught us thoroughly. I was taught it as the very foundation of my work. Old Dr. Hawes would say to us that when we would get a mouth in good order we must go "Dunning" it. Dr. Smith does not make it clear why he objects to the engine in this work. The reason is this: the deposits of tartar and other deleterious matter which may cause the loss of the teeth, especially the loss by absorption of the gum, lies just underneath the edge of the gum. This could be reached by the stick and pumice, and this method, so far as he knew, was the only method by which that part of the tooth where the deposit lies could be reached. When we remove this tartar by means of the scaler we may consider the tooth pretty thoroughly cleaned, but we forget the microscopical particles still remaining that form the nuclei for the redeposition of tartar. Hence the necessity for polishing under the gums. Dr. Kimball felt that Dr. Smith had failed to make his reasons for this work clear.

Dr. Evans had been very much interested. He did not think he could do more than to mention a little incident that came right home to him. As a matter of experiment he had decided to pursue a thorough system of cleansing in his daughter's mouth. She had a beautiful set of teeth, and he had cleansed them regularly at intervals of a month up to last year. The result was that up to then she had never developed any approximal cavities and had no fillings at all with the exception of a few in the crowns of the molars. Last year, through pressure of business and professional work he had neglected it. As she was going to Europe for a year or two, he recently carefully examined her teeth, and much to his astonishment and regret he found the approximal sides affected in nearly a dozen places, with cavities or superficial decay. He was now urging upon his patients the necessity of frequent cleansing, especially in cases of pyorrhœa. He even insisted upon this, to the extent of giving them up as patients if they would not comply.

Dr. J. B. Locherty was very much pleased with Dr. Bryan's paper, particularly the suggestion regarding the use of nitrate of silver and iodine. Dr. Locherty would also recommend dioxygen as an agent in this connection as being of great assistance in removing the green stain on children's teeth. His method was to adjust the rubber dam, apply the dioxygen and rub it over the teeth with a large heated burnisher, then polish with orange-wood sticks and pumice.

Dr. F. Milton Smith stated that it seemed to him that the whole thing was a matter of thoroughness. He could not see that there were any very new things suggested either by the paper of the evening or by Dr. Smith, of Philadelphia. It seemed to him that if Dr. Smith had announced that we were at times a little careless and that we ought to be more thorough, he would have hit the nail on the head. To this charge he would plead guilty. However, he was improving. From reading journals of an earlier date he was impressed with the fact that there had been thorough men previous to this time. He believed there were thorough men outside of Philadelphia and this side of Switzerland. He was not sure but there were some connected with the Institute of Stomatology.

He suggested that a few years ago Dr. Bonwill, of Philadelphia, gave just such an exhibition in his office as has been given by Dr. Smith. As he remembered it, the universal opinion of the men who visited Dr. Bonwill's office at that time was that the mouths exhibited were in perfect condition. Dr. Smith remembered the suggestion that Dr. Bonwill insisted that his patients come frequently to his office that he might examine their mouths and point out where they had failed to thoroughly cleanse their teeth.

Regarding the suggestion of Dr. Kimball, of polishing with a stick and pumice down under the gum, he had in mind a paper read fourteen years ago by Dr. Geo. S. Allan, and published in the INTERNATIONAL DENTAL JOURNAL of December, 1889, calling attention to the importance of securing a smooth, polished surface of the neck of the tooth just below the gum line, whether that surface was covered with tartar or not. Dr. Allan, in this paper, laid great emphasis upon the damage done the minute glands next the neck of the tooth through this roughness, causing them to throw out secretions which, he thought, were exceedingly harmful to the teeth and surrounding tissues.

Regarding people not being willing to pay for "these little things," Dr. Smith thought that the habit dentists have of rendering bills in the way they do—one gold filling, so much, and one gutta-percha filling, so much—was all wrong. People should be taught to pay for professional services.

Dr. Smith mentioned a case in point, illustrating the good results of constant care by the dentist. It was that of a young man (now twenty-eight years of age) who came to him twenty years ago. His parents came into his hands at the same time, both being affected with pyorrhœa, the father having lost half his teeth at thirty. The mother insisted that the child should go to Dr. Smith whenever he was sent for, and as a result his teeth to-day are practically perfect and free from any disease of the gum or sockets. Dr. Smith thought it right and proper to send for patients. The best and most appreciative patients he had were those who responded to these notices.

In connection with the use of pumice for polishing Dr. Bogue stated that we use this instead of chalk, because it floats on water and will float out while chalk will not.

Dr. Evans stated that in pyorrhœa cases especially it was his custom, instead of notifying them, to make another engagement then and there, notice of that engagement to be sent the patient one week previous to the date. Since he had adopted that plan things had been working very smoothly.

Dr. Swift had tried this same method mentioned by Dr. Evans, and had found it to work excellently well. In connection with hydrogen peroxide, he would recommend the preparation put up by the American Chemical Company as being very stable, and containing less than one-twentieth per cent. acidity. It comes in five-pound bottles at one dollar and fifty cents per bottle.

Dr. Dailey spoke of the necessity of an alkaline condition of the mouth in order to dissolve this deposit around the necks of the teeth. In an acid saliva micro-organisms developed very rapidly, but if alkaline the spores will not develop. He thought we should take this home with us. Dr. Williams had shown teeth of animals that had commenced to decay and then stopped, showing that at some period of drought or migration there was a condition of malnutrition, but when the animal had resumed his normal environment the decay stopped. An alkaline condition of the mouth was necessary. The majority of civilized people had an acid saliva. Dr. Dailey strongly advocated the use of a solution of bicarbonate of soda to restore the alkalinity of the saliva.

Adjourned.

FRED. L. BOGUE, M.D., D.D.S.,
*Editor The New York Institute of Stomatology.*

## Figures and Tables

**Fig. 1. f1:**
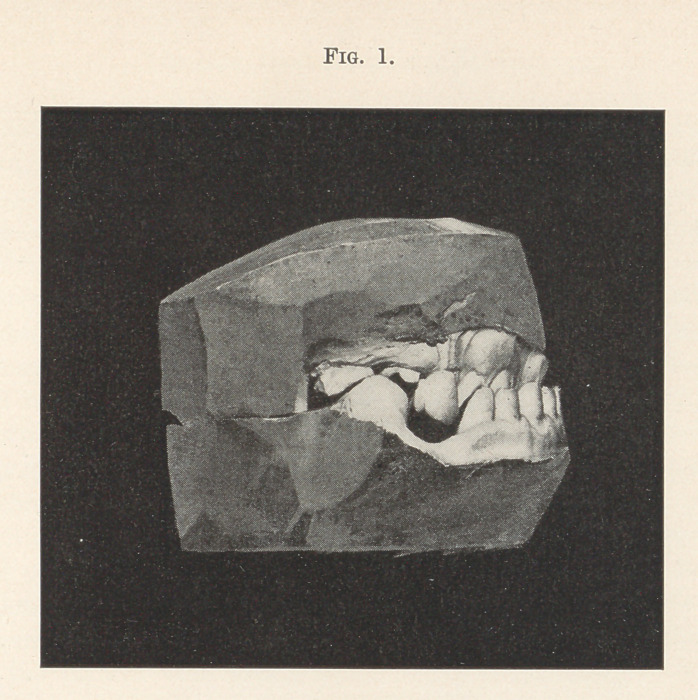


**Fig. 2. f2:**